# Metabolic profiling of petroleum-degrading microbial communities incubated under high-pressure conditions

**DOI:** 10.3389/fmicb.2023.1305731

**Published:** 2023-12-22

**Authors:** Jinbo Xu, Lu Wang, Weifeng Lv, Xinmin Song, Yong Nie, Xiao-Lei Wu

**Affiliations:** ^1^School of Earth and Space Sciences, Peking University, Beijing, China; ^2^State Key Laboratory of Enhanced Oil and Gas Recovery, Research Institute of Petroleum Exploration and Development, Beijing, China; ^3^College of Engineering, Peking University, Beijing, China; ^4^Institute of Ecology, Peking University, Beijing, China

**Keywords:** oil reservoir, metagenomics, pressure, methanogenesis, sulfate-reducing

## Abstract

While pressure is a significant characteristic of petroleum reservoirs, it is often overlooked in laboratory studies. To clarify the composition and metabolic properties of microbial communities under high-pressure conditions, we established methanogenic and sulfate-reducing enrichment cultures under high-pressure conditions using production water from the Jilin Oilfield in China. We utilized a metagenomics approach to analyze the microbial community after a 90-day incubation period. Under methanogenic conditions, Firmicutes, Deferribacteres, Ignavibacteriae, Thermotogae, and Nitrospirae, in association with the hydrogenotrophic methanogen Archaeoglobaceae and acetoclastic *Methanosaeta*, were highly represented. Genomes for *Ca*. Odinarchaeota and the hydrogen-dependent methylotrophic *Ca*. Methanosuratus were also recovered from the methanogenic culture. The sulfate-reducing community was dominated by Firmicutes, Thermotogae, Nitrospirae, *Archaeoglobus*, and several candidate taxa including *Ca*. Bipolaricaulota, *Ca*. Aminicenantes, and Candidate division WOR-3. These candidate taxa were key pantothenate producers for other community members. The study expands present knowledge of the metabolic roles of petroleum-degrading microbial communities under high-pressure conditions. Our results also indicate that microbial community interactions were shaped by syntrophic metabolism and the exchange of amino acids and cofactors among members. Furthermore, incubation under *in situ* pressure conditions has the potential to reveal the roles of microbial dark matter.

## 1 Introduction

Petroleum reservoirs are extreme environments with high pressure, high salinity, and high temperature (Youssef et al., 2009). Recent studies have shown that various microbes colonize these unique habitats and that they have a significant influence on the quality and recovery of oil (Van Hamme et al., 2003; Li et al., 2017; Varjani and Gnansounou, 2017). On the positive side, the microbes in petroleum reservoirs produce various metabolites (gases, acids, biopolymers, and biosurfactants) that may enhance oil recovery (Bachmann et al., 2014). However, negatively, some organisms may degrade hydrocarbons, altering oil components, and resulting in the production of heavy oil (Head et al., 2003). Therefore, it is important to understand the metabolism and functions of microbial communities in petroleum reservoirs.

Many reports have demonstrated the biodegradation of petroleum hydrocarbons under nitrate-, ferric-, and sulfate-reducing conditions (Shin et al., 2019; Zhang et al., 2019; Pavlova et al., 2022); when these electron acceptors are depleted, the hydrocarbons can be further converted into methane and carbon dioxide (Jones et al., 2008; Mbadinga et al., 2012; Ma et al., 2017; Liu et al., 2019). Sulfate-reducing prokaryotes (SRPs) were the first microorganisms reported in oil reservoirs (Bastin et al., 1926). They can utilize hydrocarbons, volatile fatty acids (VFAs), and H_2_ while reducing sulfate to produce sulfide. This process contributes to reservoir corrosion and souring, increased refining costs, and health risks (Li et al., 2017; Vigneron et al., 2017; Singh and Choudhary, 2021). Although there have been studies on the diversity and distribution of SRPs in oil reservoirs (Guan et al., 2013; Fan et al., 2017), information on the interactions between SRPs and other community members during petroleum degradation is still limited. Methanogenesis is another important process in subsurface petroleum reservoirs. Geochemical analyses of well data from the Wilcox Group in Louisiana and the Olla Oil Field in Louisiana, USA, supported the relationship between methanogenesis and petroleum biodegradation (Shelton et al., 2016; Tyne et al., 2021). Microbial-enhanced oil recovery (MEOR), which involves methanogenic crude oil biodegradation, is considered an alternative energy source that can contribute to the global energy profile (Kryachko, 2018). Therefore, it is essential to study the methanogenic biogeochemistry process by simulating the real environment of oil reservoirs as accurately as possible.

The physical and chemical parameters of petroleum reservoirs (e.g., temperature, salinity, nutrients, and electron acceptors) vary and may influence the composition and function of the native microbial communities (Gittel et al., 2009; Piceno et al., 2014; Wang et al., 2019a; Zhou et al., 2019). However, the majority of laboratory enrichment incubations are carried out under ambient pressure, ignoring that petroleum reservoirs are high-pressure environments. Pressure affects the structure of cellular components, including DNA, protein, and cell membranes, as well as cellular processes such as DNA/RNA synthesis, translation, membrane fluidity, motility, cell division, and nutrient uptake (Oger and Jebbar, 2010; Mota et al., 2013; Picard and Daniel, 2013; Jebbar et al., 2015). More recently, it has been confirmed that pressure can restructure deep-sea hydrocarbon-degrading microbial communities (Fasca et al., 2018; Calderon et al., 2019). However, there have been limited studies on microorganisms in oil reservoirs under high-pressure conditions. It has been demonstrated that syntrophic acetate oxidation (SAO) coupled with hydrogenotrophic methanogenesis is the primary methanogenic pathway in a high-temperature petroleum reservoir. This was shown using natural microcosms consisting solely of oil-field samples under conditions of 55°C and 5 MPa, simulating the *in situ* subsurface oil reservoir environment (Mayumi et al., 2011). More interestingly, it was found that high CO_2_ conditions invoke acetoclastic methanogenesis in place of SAO coupled with hydrogenotrophic methanogenesis under incubation at 55°C and 5 MPa (Mayumi et al., 2013). In another study, high-pressure bioreactors were used to simulate *in situ temperature* (55°C) and oil-bearing sand, without applying external pressure. Analysis of archaeal and bacterial 16S rRNA gene sequences revealed a shift in the predominant methanogenic community from methylotrophic methanogens to thermophilic hydrogenotrophic methanogens after long-time incubation in the bioreactor (Xu et al., 2019). These studies have provided valuable insights into *in situ* incubation. However, while the 16S rRNA analyses have expanded our knowledge of the methanogenic processes over time of incubation, they are limited in providing a deeper understanding of microbial metabolic properties. As such, it is necessary to conduct metagenomic and/or metatranscriptomic sequencing and examine the genes involved in anaerobic hydrocarbon biodegradation pathways in order to clarify the roles of microbes.

In the present study, we conducted laboratory-scale high-pressure incubations with production water from the Jilin Oilfield under sulfate-reducing and methanogenic conditions. We then sequenced the metagenome of the microbial communities after incubation and assembled genomic bins. The study aimed to clarify the characterization and ecological role of anaerobic petroleum-degrading microbes under pressure in oil reservoirs.

## 2 Materials and methods

### 2.1 High-pressure incubation, DNA extraction, and library preparation

Production water was collected from the Jilin Oilfield and stored in stainless steel reactors (1 L) at 5 MPa. The samples were transported to the laboratory within 48 h, at which point the pressure was immediately increased to 12 MPa. After adding fumarate (10 mM, 90 mL) or sulfate (10 mM, 90 mL), the reactors were incubated statically for 90 days at 60°C. DNA was extracted and purified using the FastDNA SPIN Kit (MP Biomedicals) following the manufacturer's protocols. The genomic libraries were sequenced on a HiSeq 2500 platform (Illumina, San Diego, CA, USA) with a 2 × 150-bp paired-end run at Magigene, Guangzhou.

### 2.2 Metagenome assembly and binning

The raw reads were trimmed using sickle (v1.33, http://github.com/najoshi/sickle) with the “pe” mode and default settings. The trimmed reads were assembled using SPAdes v3.14.0 (metaSPAdes mode) with the default kmers: “21, 33, 55, 77” (Nurk et al., 2017). The contigs were then binned using MetaWRAP v1.2.1 (Uritskiy et al., 2018), which includes Metabat2, Maxbin2, and CONCOCT. Results from the three binning tools were combined using MetaWRAP and de-contaminated using the “outliers” method of RefineM v0.1.2. The completeness and contamination of all MAGs were estimated with CheckM (Parks et al., 2015; Uritskiy et al., 2018).

### 2.3 Genome analysis

The MAGs were taxonomically assigned using the GTDB-Tk (v1.3.0) tool with the Genome Taxonomy database (GTDB, release202) (Chaumeil et al., 2020). The average amino acid identity (AAI) values were calculated by EzAAI v1.1 (Kim et al., 2021). Relative abundance for each MAG was calculated by CoverM with parameters “-min-read-percent-identity 0.95 -min-read-aligned-percent 0.75” (v0.6.1, https://github.com/wwood/CoverM). Protein-coding genes were predicted using prodigal (v2.6.3) with -p meta parameters (Hyatt et al., 2010). The MAGs were then annotated using eggnog-mapper v2.0.1 in the EggNOG database (Huerta-Cepas et al., 2017, 2019) and GhostKOALA for KEGG pathways (Kanehisa and Goto, 2000). Genes involved in anaerobic hydrocarbon degradation were searched against a previously curated database using BlastP (e-value, 1e-10; identity, 30%) (Dong et al., 2019). Hydrogenases were identified by DIAMOND BlastP via a comparison with HydDB (e-value, 1e-50; qcover, 90%; identity, 50%) (Ju and Zhang, 2015; SøNdergaard et al., 2016).

### 2.4 Phylogenetic analysis

For taxonomic classification, 400 conserved protein sequences in MAGs and reference genomes were extracted, aligned, and concatenated by PhyloPhlAn v3.0.60 (Segata et al., 2013). For phylogenetic analysis of functional genes, amino acid sequences were downloaded from GenBank and aligned using Muscle v3.8 (Edgar, 2004) with the default parameters, followed by refinement using trimAl 1.4 (Capella-Gutierrez et al., 2009) with the option “automated1”. The trees were built using the IQ-Tree method with the model MFP with 1,000 bootstrap replicates (Lam-Tung et al., 2015). Finally, the trees were visualized and annotated using the online tool iTOL (Letunic and Bork, 2016).

## 3 Results and discussion

### 3.1 Metagenomic profiling

**Figure 1:**
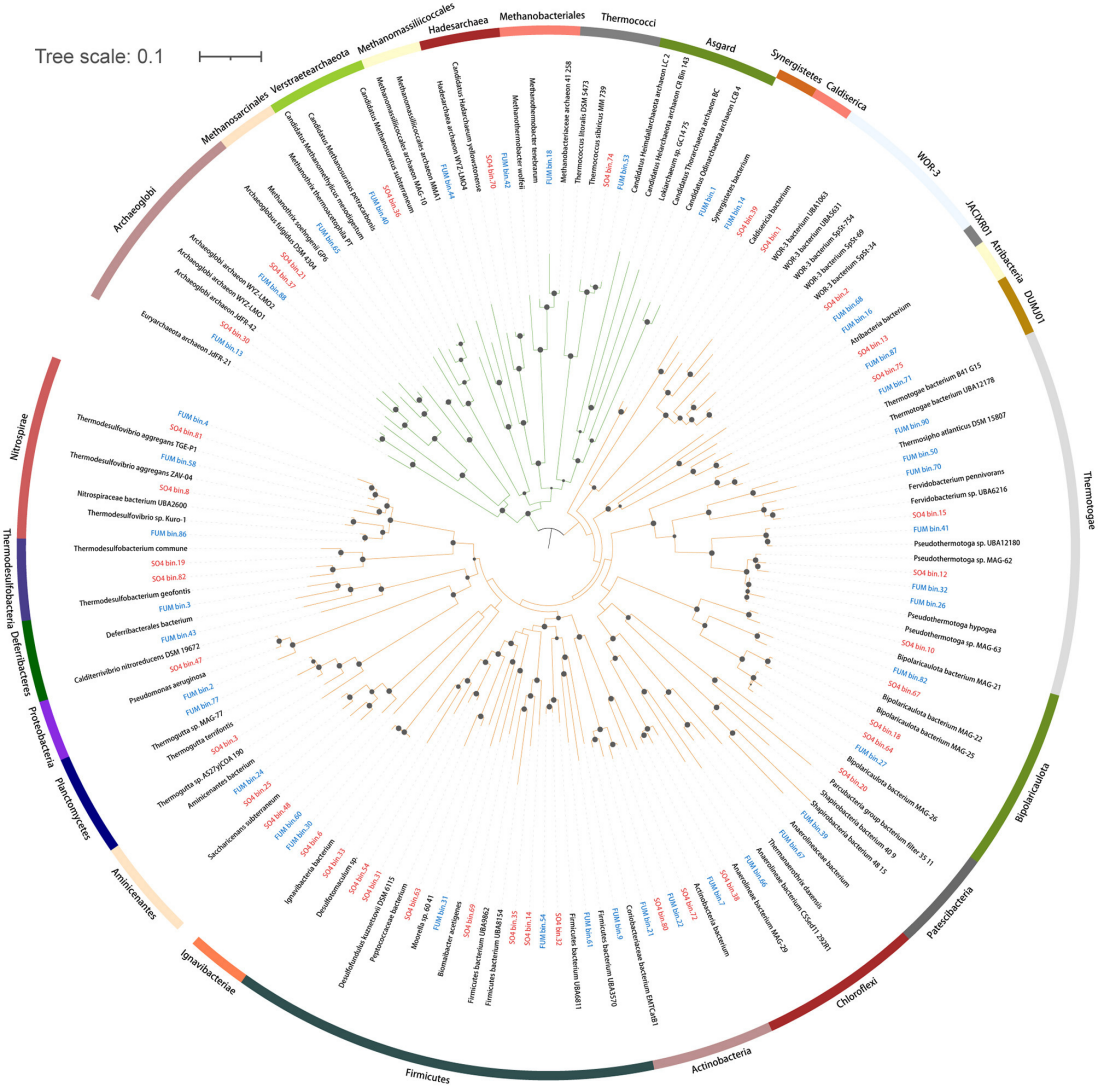
Phylogenetic tree of 65 medium- to high-quality bacterial MAGs and 15 medium- to high-quality archaeal MAGs assembled from the FUM group (blue) and the SO4 group (red). Green: Archaea. Orange: Bacteria. The maximum-likelihood tree was built based on 400 conserved protein sequences using IQ-Tree with the best-fit model and 1,000 bootstrap replicates. Circles represent bootstrap values >70%.

We recovered 42 and 38 medium- to high-quality MAGs (completeness >50% and contamination < 10%) from an oil-degrading community incubated with fumarate (FUM, methanogenic conditions) and sulfate (SO4, sulfate-reducing conditions), respectively (Supplementary Table S1). For FUM and SO4, 68.9% and 74.7% of the reads were mapped to the respective MAGs. A total of 80 MAGs were taxonomically assigned to 20 bacterial phyla and 3 archaeal phyla (Figure 1, Supplementary Table S1). Firmicutes (12 MAGs) and Thermotogae (9 MAGs) were the dominant bacterial taxa. For Archaea, most MAGs were assigned to the phylum Euryarchaeota. Two MAGs were classified as Verstraetearchaeota, and one was classified as Asgard. There were MAGs specific to each enrichment condition, such as Dictyoglomi, Deferribacteres, Synergistetes, Coprothermobacterota, and Armatimonadetes from the FUM group, and Caldiserica, Thermodesulfobacteria, and Atribacteria from the SO4 group. Based on the taxonomy and metabolic potential of these MAGs, we analyzed the main metabolic groups of microorganisms under the different electron acceptors.

### 3.2 Thermophilic methanogenic community

The taxonomic analyses of the metagenome, based on searches against the NR database, revealed the community structure (Supplementary Figure S1): Euryarchaeota (30.9% of the metagenome), Firmicutes (22.4%), Thermotogae (10.7%), Chloroflexi (4.6%), Nitrospirae (2.8%), Ignavibacteriae (1.6%), Deferribacteres (1.4%), and Proteobacteria (1.3%).

#### 3.2.1 Hydrocarbon degradation

**Figure 2:**
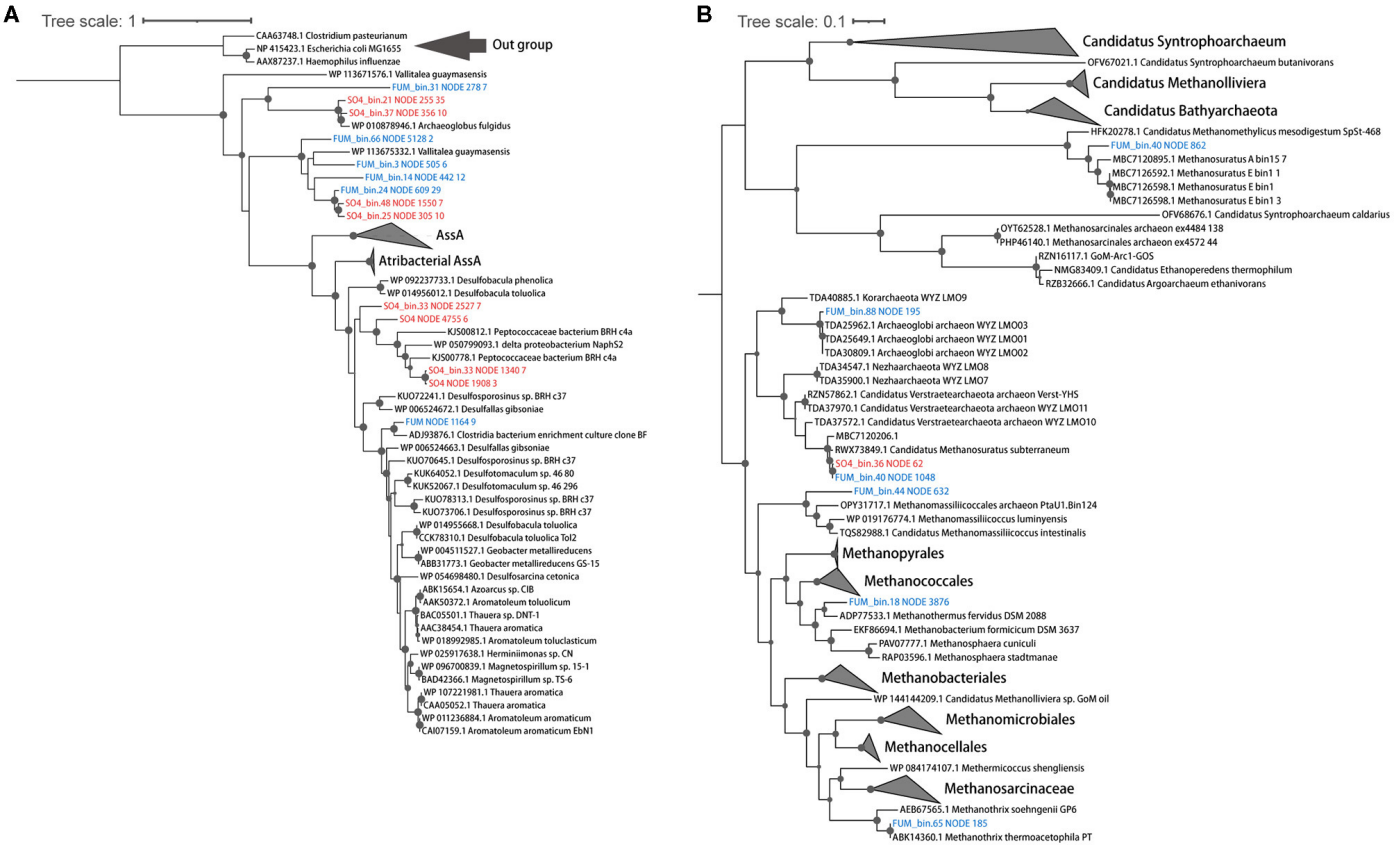
Phylogenetic affiliations of the putative FaeA **(A)** and McrA **(B)** protein sequences found in the FUM group (blue) and the SO4 group (red). Bootstrap values >70% are indicated. Reference sequences of FaeA include canonical AssA, BssA, NmsA, Atribacterial AssA, and homologous putative AssA from *Vallitalea guaymasensis* L81 and *Archaeoglobus fulgidus* VC-16. Reference sequences of McrA include canonical and Verstraetearchaeota-type McrA for methane production and AcrA for ethane or butane oxidation.

**Figure 3:**
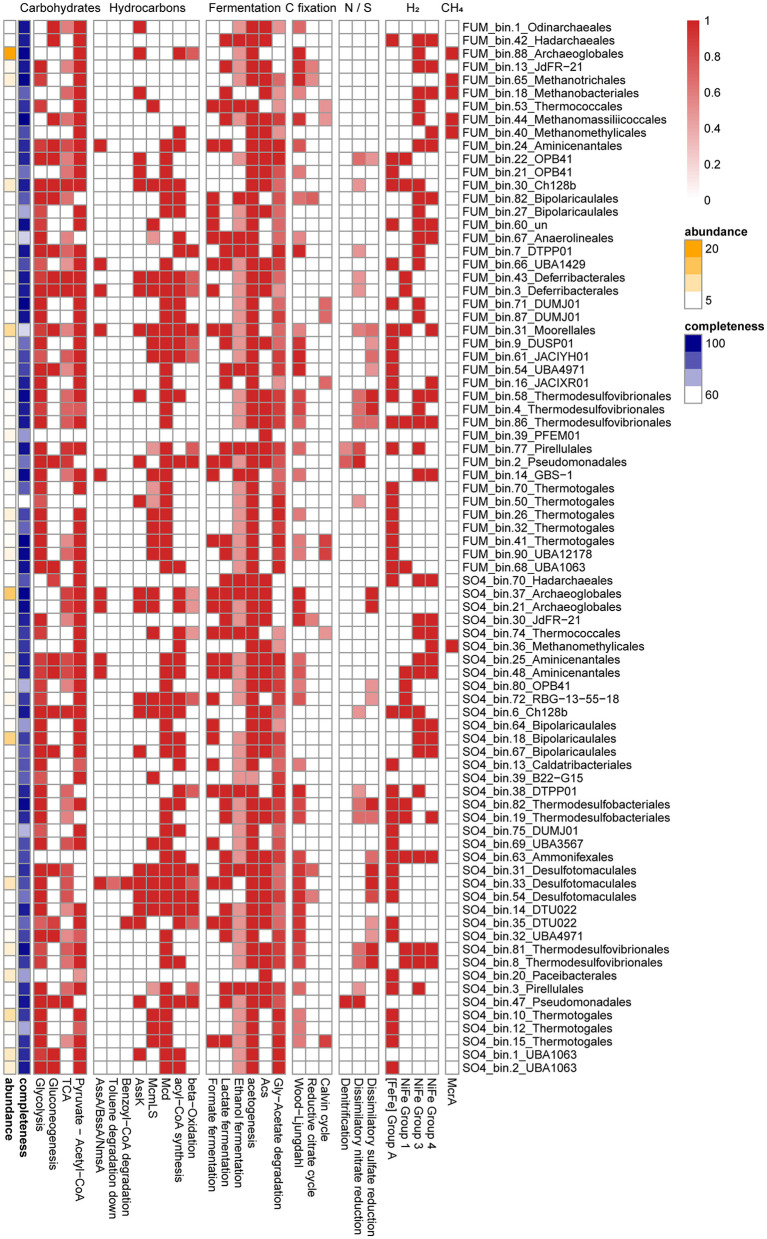
Heatmap of metabolic pathways or genes identified in the medium- to high-quality MAGs. Pathways were indicated when 50% of the genes involved in the pathway were detected. A complete list of these functional genes can be found in Supplementary Table S2.

Activation of hydrocarbons by fumarate addition is an important pathway of anaerobic hydrocarbon degradation and is mediated by fumarate-adding enzymes (FAEs), including alkyl-succinate synthases (Ass, for alkanes), benzyl succinate synthases (Bss, for alkyl-substituted benzenes), and naphthylmethyl-succinate synthases (Nms, for methylnaphthalenes) (Callaghan et al., 2010). A BssA sequence was observed in the fumarate metagenome, but it was not assigned to any of the MAGs from that enrichment condition (Figure 2A). Other FaeA sequences, which showed low homology to canonical alkyl-/arylalkyl-succinate synthetase, were found in five MAGs (Synergistetes, Aminicenantes, Deferribacteres, Firmicutes, and Chloroflexi) (Figure 2A). They were closely associated with archaea-type AssA from *Archaeoglobus fulgidus* VC-16 and *Vallitalea guaymasensis* L81, which were also described in previous studies (Khelifi et al., 2014; Dong et al., 2019). A Moorellales bin (FUM_bin.31_Moorellales), from Firmicutes proposed in the GTDB taxonomy, was the most abundant bacterial MAG, accounting for 9.9% of the total MAGs. It shared 98.3% AAI with a *Moorella* MAG (Moorella_M_bin9) recovered from thermophilic *n*-paraffin-degrading (C_21_-C_30_) cultures (Liu et al., 2020a). FUM_bin.31_Moorellales possessed a potential FaeA and its activating enzyme (AssD), as well as many of the genes required for the utilization of activated hydrocarbons. These genes include acyl-CoA synthetase/ligase (AssK), methylmalonyl-CoA mutase (McmLS), and methylmalonyl-CoA carboxyltransferase (Mcd). Subsequently, the acyl-CoA produced from alkane oxidation could be oxidized to acetyl-CoA through β-oxidation, including acyl-CoA dehydrogenase (Acd), enoyl-CoA hydratase (Ech), 3-hydroxyacyl-CoA dehydrogenase (Had), and acetyl-CoA acyltransferase (AtoB), suggesting that this Moorellales bin may play a critical role in alkane degradation (Figure 3).

It was noteworthy that FUM_bin.3_Deferribacterales (0.5% of the total MAGs), assigned to the family Flexistipitaceae from Deferribacteres in the GTDB taxonomy, possessed an archaea-type AssA and its activating enzyme (AssD). Genes for the utilization of activated hydrocarbons (AssK, McmLS, and Mcd) and β-oxidation (except acyl-CoA dehydrogenase) were also identified in the MAG (Figure 3). To date, no cultivated member of the Deferribacteres has been reported to possess the capacity for hydrocarbon degradation, but a benzoate-CoA ligase was present in a Deferribacteres bin from the Halfdan oil field in the North Sea (Vigneron et al., 2017; Shlimon et al., 2020). Based on the potential pathway in FUM_bin.3_Deferribacterales, we speculate that Deferribacteres may participate in hydrocarbon activation. Due to the low sequence similarity to known bacterial FaeA, experimental verification of whether the two species are capable of hydrocarbon degradation is still required.

#### 3.2.2 Fermentation of intermediate metabolites and production of acetate and hydrogen

FUM_bin.43_Deferribacterales (1.2% of the total MAGs), assigned to the family Flexistipitaceae from Deferribacteres, and FUM_bin.9_DUSP01 (1.1%), assigned to the candidate order DUSP01 from Firmicutes, encoded long-chain acyl-CoA synthetase (FadD), Ech, Had, and AtoB, suggesting that they participated in fatty acid degradation (Figure 3).

In addition to hydrocarbons, detrital biomass may be an important substrate. FUM_bin.30_Ch128b, assigned to the candidate genus Ch128b from Bacteroidota in GTDB, was the second most abundant bacterial MAG (5.0%). Members of the Ignavibacteria are able to grow on various carbohydrates with fermentative metabolism (Kadnikov et al., 2020b). Consistent with this, FUM_bin.30_Ch128b contained a complete set of genes for the Embden–Meyerhof pathway (EMP) of glycolysis, as well as pyruvate ferredoxin oxidoreductase (Por) for converting pyruvate to acetyl-CoA. The presence of ADP-forming acetyl-CoA synthetase (AcdA) and group 3b, 3d NiFe-hydrogenases indicated that H_2_ and acetate were produced as fermentation products (Figure 3). Interestingly, a nearly complete genome of *Ca*. Odinarchaeota (FUM_bin.1_Odinarchaeales, 0.2%) was recovered from the FUM group, with a 97.7% AAI to a *Ca*. Odinarchaeota, an archaeal MAG recovered from the Shengli Oilfield in China (Liu et al., 2021). *Ca*. Odinarchaeota has been predicted to be almost exclusively present in high-temperature habitats (Zaremba-Niedzwiedzka et al., 2017), and it coincides with the higher incubation temperature in our culture. The complete pathway of gluconeogenesis and partial TCA cycle were present in FUM_bin.1_Odinarchaeales, and the former uses lactate, pyruvate, or amino acids as a precursor for glucose production (Grasmann et al., 2019). Within FUM_bin.1_Odinarchaeales, there were genes responsible for converting aspartate, asparagine, and glutamate to TCA cycle intermediates, which then entered the gluconeogenesis pathway. FUM_bin.1_Odinarchaeales also encoded several enzymes for the Wood–Ljungdahl (WL) pathway and AcdA, indicating its potential to ferment organic substrates into acetate and CO_2_ (Figure 3) (Spang et al., 2019).

Syntrophic acetate oxidation (SAO) is a significant process during hydrocarbon degradation and is often associated with hydrogenotrophic methanogenesis. Thermotogae, which are capable of fermenting complex substrates, are frequently detected in high-temperature oil reservoirs (Li et al., 2017). Two Thermotogae MAGs (FUM_bin.26_Thermotogales, 3.7%; FUM_bin.90_UBA12178, 2.7%) encoded the complete pathways for metabolizing glucose and producing acetate. While *Pseudothermotoga* MAG FUM_bin.26_Thermotogales contained an incomplete acetyl-CoA synthase/CO dehydrogenase (Acs/Codh) for the oxidative WL pathway, it did possess a complete glycine cleavage system and tetrahydrofolate pathway for acetate oxidation previously proposed in a syntrophic acetate degrader (Figure 3) (Nobu et al., 2015a; Zhu et al., 2020). The generated NADH could be reoxidized by a proton-translocating ferredoxin:NAD^+^ oxidoreductase (Rnf) present in the genome (Supplementary Table S2) (Nobu et al., 2015a; Manzoor et al., 2018). The electron-confurcating FeFe-hydrogenases (ECHyd) then drive Fd_red_ and NADH oxidation for H_2_ generation. In addition, the *Thermodesulfovibrio* MAG FUM_bin.58_Thermodesulfovibrionales (1.2%) possessed the complete WL pathway and FeFe-hydrogenases (Figure 3). Although it encoded an incomplete Rnf complex, a FAD/NAD(P)-binding protein was identified, which shared 40% sequence identity with a potential ion-translocating ferredoxin:NADH oxidoreductase (Ifo) in *Syntrophorhabdus aromaticivorans* strain UI (Nobu et al., 2015b). It was previously reported that *Thermodesulfovibrio* could form a syntrophic relationship with hydrogenotrophic methanogens and may be involved in SAO (Sekiguchi et al., 2008; Yang et al., 2016; Zheng et al., 2019). Together, the presence of an acetate oxidation pathway, reverse electron transport complex, and electron-confurcating hydrogenase suggested that FUM_bin.26_Thermotogales and FUM_bin.58_Thermodesulfovibrionales performed SAO during hydrocarbon degradation.

On the other hand, homoacetogenesis via the WL pathway may exist in methanogenic oil-degrading communities. The candidate genus JdFR-21 from Archaeoglobi (FUM_bin.13_JdFR-21), which accounted for 1.8% of total MAGs, contained a complete archaeal WL pathway and AcdA, indicating that it may produce acetate for SAO bacteria and acetoclastic methanogens (Figure 3).

#### 3.2.3 Production of methane

We recovered five high-quality methanogenic MAGs from the FUM group, including *Methanosaeta* (FUM_bin.65_Methanotrichales), *Methanothermobacter* (FUM_bin.18_Methanobacteriales), *Methanomassiliicoccus* (FUM_bin.44_Methanomassiliicoccales), *Ca*. Methanosuratus (FUM_bin.40_Methanomethylicales), and Archaeoglobaceae (FUM_bin.88_Archaeoglobales). Archaeoglobaceae MAG (24.2%) and *Methanosaeta* MAG (4.1%) were dominant among the methanogens. A phylogenetic tree was built to show the evolutionary relationships of the alpha subunit of the methyl-coenzyme M reductase complex (McrABG or MCR) protein sequence retrieved from these MAGs (Figure 2B). FUM_bin.65_Methanotrichales, FUM_bin.18_Methanobacteriales, and FUM_bin.44_Methanomassiliicoccales encoded a canonical McrA, which clustered with those of the phylum Euryarchaeota, including *Methanothermobacter, Methanosaeta*, and *Methanomassiliicoccus* (Figure 2B). McrA in FUM_bin.88_Archaeoglobales was between the canonical and divergent McrA, which is a Verstraetearchaeota-type McrA, according to Wang et al. (2020) (Figure 2B). FUM_bin.40_Methanomethylicales contained a Verstraetearchaeota-type McrA but also a highly divergent McrA related to *Ca*. Argoarchaeum, which has been confirmed to be an anaerobic ethane-oxidizing archaeon (Figure 2B) (Chen S. C. et al., 2019).

FUM_bin.88_Archaeoglobales shared 82.2% AAI with WYZ-LMO2 (Wang et al., 2019b), which is a potential hydrogenotrophic methanogen. In addition to the WL pathway and tetrahydromethanopterin S-methyltransferase (MTR), FUM_bin.88_Archaeoglobales also contained (methyl-Co(III) methanol/glycine betaine-specific corrinoid protein):coenzyme M methyltransferase (MtaABC) for methylotrophic methanogenesis. Heterodisulfide reductase (HdrABC) and F_420_-nonreducing hydrogenase (MvhADG) are believed to regulate the oxidation of hydrogen while driving the endergonic reduction of ferredoxin and the exergonic reduction of CoM-S-S-CoB in an electron-bifurcating reaction during hydrogen-dependent methanogenesis. However, the HdrB subunit, which catalyzes the reduction of CoM-S-S-CoB, was not found in the MAG. Indeed, a homologous heterodisulfide reductase, HdrD, may replace HdrB in the Hdr/Mvh complex. It is also detected in another Archaeoglobi genome and a Korarchaeota genome (McKay et al., 2019; Liu et al., 2020b). Moreover, although the genome lacked HdrE, it contained a gene cluster encoding the NADH-ubiquinone oxidoreductase complex (Nuo) without NuoEFG, which showed high sequence similarity to Fpo. This Fpo-like complex may couple with HdrD to directly receive electrons from Fd_red_ with the reduction of CoM-S-S-CoB and establish a membrane potential, as suggested in the H_2_-dependent methylotrophic *Methanomassiliicoccus luminyensis* (Lang et al., 2015; Kroeninger et al., 2016). Hence, FUM_bin.88_Archaeoglobales may utilize hydrogen for methanogenesis (Supplementary Figure S2A). However, these hypotheses require further verification through the cultivation of representative organisms.

FUM_bin.40_Methanomethylicales (0.1%) clustered within *Methanosuratus*, a genus belonging to the Verstraetearchaeota (the phylum Thermoproteota and order Methanomethylicales in GTDB). Like other members of this phylum, a canonical MCR and the genes required for methylotrophic methanogenesis (*mtaABC*) were present in the genome (Vanwonterghem et al., 2016; Borrel et al., 2019). In addition to the canonical MCR, there was also a highly divergent MCR (alkyl-coenzyme M reductase, ACR) in FUM_bin.40_Methanomethylicales (Chen S. C. et al., 2019), related to those of ethane-degrading archaea *Ca*. Argoarchaeum ethanivorans and *Ca*. Ethanoperedens thermophilum (Chen S. C. et al., 2019; Hahn et al., 2020). However, the lack of a WL pathway indicated that it could not completely metabolize short-chain alkanes to CO_2_. However, with the presence of AcdA in FUM_bin.40_Methanomethylicales, we infer that it may produce acetate during short-chain alkane oxidation (Supplementary Figure S2B).

### 3.3 Thermophilic sulfate-reducing community

The taxonomic analyses of the metagenome based on searches against the NR database revealed the community structure (Supplementary Figure S1): Euryarchaeota (24.8% of metagenome), Firmicutes (17.8%), Thermotogae (13.6%), Chloroflexi (4.6%), *Ca*. Bipolaricaulota (8.5%), Nitrospirae (5.7%), *Ca*. Hydrothermae (4.9%), *Ca*. Aminicenantes (3.7%), Actinobacteria (2.8%), Proteobacteria (1.3%), and Patescibacteria (1.2%).

#### 3.3.1 Hydrocarbon degradation

For anaerobic hydrocarbon degradation, AssA, whose role in alkane degradation has been confirmed in *Archaeoglobus fulgidus* DSM 4304 (Khelifi et al., 2014), was also found in the *Archaeoglobus* MAGs (SO4_bin.37_Archaeoglobales, 14.4%) (Figure 2A). SO4_bin.37_Archaeoglobales also contained genes for the activating enzyme AssD and the degradation of activated hydrocarbons (AssK, McmLS, and Mcd). However, it only encoded Acd and AtoB for incomplete β-oxidation (Figure 3). Therefore, fatty acids may be further degraded by other community members, such as SO4_bin.72_RBG-13-55-18 (from the candidate order RBG-13-55-18 of Actinobacteria in GTDB, 2.1%) and SO4_bin.14_DTU022 (from the candidate family UBA8154 of Firmicutes in GTDB, 0.5%), which contained genes for β-oxidation (Figure 3).

SO4_bin.33_Desulfotomaculales (6.8%), assigned to the candidate family Nap2-2B of Firmicutes in the GTDB taxonomy, shared 64.5% AAI with Peptococcaceae SCADC (from Nap2-2B in GTDB), which contained a putative gene encoding AssA (Tan et al., 2014). SO4_bin.33_Desulfotomaculales may also be able to degrade hydrocarbons, given the putative NmsA in its genome. Phylogenetic analysis of the NmsA gene showed that it clustered with NmsA in the Peptococcaceae bacterium BRH c4a and the Deltaproteobacteria strain NaphS2 (Figure 2A). Strain NaphS2 was isolated from marine sediment in anoxic sulfate-rich medium and could grow on naphthalene, 2-naphthoate, benzoate, acetate, and pyruvate by sulfate reduction (Galushko et al., 1999). After activation by fumarate addition, 2-naphtylmethylsuccinic acid was further degraded by BnsA-H (Meckenstock et al., 2016). However, BnsA-H was not detected in SO4_bin.33_Desulfotomaculales. Instead, it encoded BbsBDEFGH for the utilization of benzyl succinate. For the degradation of benzoyl-CoA, ATP-independent benzoyl-CoA reductase (BamBC), cyclohexa-1,5-dienecarbonyl-CoA hydratase (Dch), 6-hydroxycylohex-1-ene-1-carboxyl-CoA dehydrogenase (Had), and 6-oxocyclohex-1-ene-1-carbonyl-CoA hydrolase (Oah) were identified in SO4_bin.33_Desulfotomaculales (Figure 3). In addition, genes for phenol degradation were also detected in this MAG, i.e., flavin prenyltransferase (UbiX), 4-hydroxybenzoate-CoA ligase (HbaA), and 4-hydroxybenzoyl-CoA reductase subunit alpha (HcrA) (Supplementary Table S2). Together, these results strongly suggest that SO4_bin.33_Desulfotomaculales may utilize aromatic compounds as substrates.

#### 3.3.2 Sulfate reduction

A complete sulfate reduction pathway, including sulfate adenylyltransferase (Sat), adenylylsulfate reductase (AprAB), and dissimilatory sulfite reductase (DsrAB), was found in seven MAGs from *Archaeoglobus, Thermodesulfobacterium* (Thermodesulfobacteria, the phylum Desulfobacterota in GTDB), *Thermodesulfovibrio* (Nitrospirae), *Desulfofundulus* (Firmicutes), and the candidate family Nap2-2B (Firmicutes) (Figure 3). In addition to SO4_bin.37_Archaeoglobales and SO4_bin.33_Desulfotomaculales, which were identified as key hydrocarbon degraders, SO4_bin.81_Thermodesulfovibrionales (*Thermodesulfovibrio*) also accounted for a large proportion (4.2%) of total MAGs. Although we speculated that the *Thermodesulfovibrio* MAG in the methanogenic community performed SAO, *Thermodesulfovibrio* could also grow through the reduction of sulfate with hydrogen and formate when sulfate was available (Yang et al., 2016). Group 1 NiFe-hydrogenases were found in this MAG, which indicated that SO4_bin.81_Thermodesulfovibrionales may utilize hydrogen, which was produced by the fermentative members in a syntrophic relationship.

#### 3.3.3 Fermentative metabolism of candidate phyla

We reconstructed several MAGs assigned to *Ca*. Aminicenantes, *Ca*. Atribacteria, *Ca*. Bipolaricaulota, and Candidate division WOR-3, which were also found in oil reservoirs or oil-impacted environments in previous studies (Zhou et al., 2012; Hu et al., 2016; Toth and Gieg, 2018; Kadnikov et al., 2020a). The presence of genes for glycolysis, Por, AcdA, and evolving hydrogenases (group 4 NiFe-hydrogenases, FeFe-hydrogenases) in *Ca*. Bipolaricaulota (SO4_bin.18_Bipolaricaulales, 11.8%), Candidate division WOR-3 (SO4_bin.1_UBA1063, 6.1%; SO4_bin.2_UBA1063, 4.2%), and *Ca*. Aminicenantes (SO4_bin.25_Aminicenantales, 1.8%; SO4_bin.48_Aminicenantales, 1.1%) indicated their role in carbon and hydrogen cycling (Figure 3). Interestingly, we detected a complete glycine cleavage system for SAO, Rnf complex, and ECHyd in SO4_bin.2_UBA1063 (Supplementary Table S2), suggesting that it may be a syntrophic bacterium. Moreover, a Parcubacteria (OD1) genome (SO4_bin.20_Paceibacterales, 5.2%), belonging to Patescibacteria, was recovered from the sample. Parcubacteria have a small genome and possess various metabolic capabilities. However, they lack several core biosynthetic capacities, including those for nucleotides, lipids, fatty acids, and many amino acids, as such, they commonly live a symbiotic lifestyle with other microbes (Castelle et al., 2018). Many Parcubacteria can produce acetate and hydrogen via AcdA and hydrogenases (Castelle et al., 2018). However, the capabilities of glycolysis and acetate generation were absent in SO4_bin.20_Paceibacterales (Figure 3). It was not certain whether the absence was due to the incompleteness of the genome (70.82%). Despite this, SO4_bin.20_Paceibacterales had FeFe-hydrogenases (Figure 3), which were likely associated with H_2_-utilization sulfate-reducing prokaryote (SRP).

### 3.4 Metabolic interactions in microbial communities

**Figure 4:**
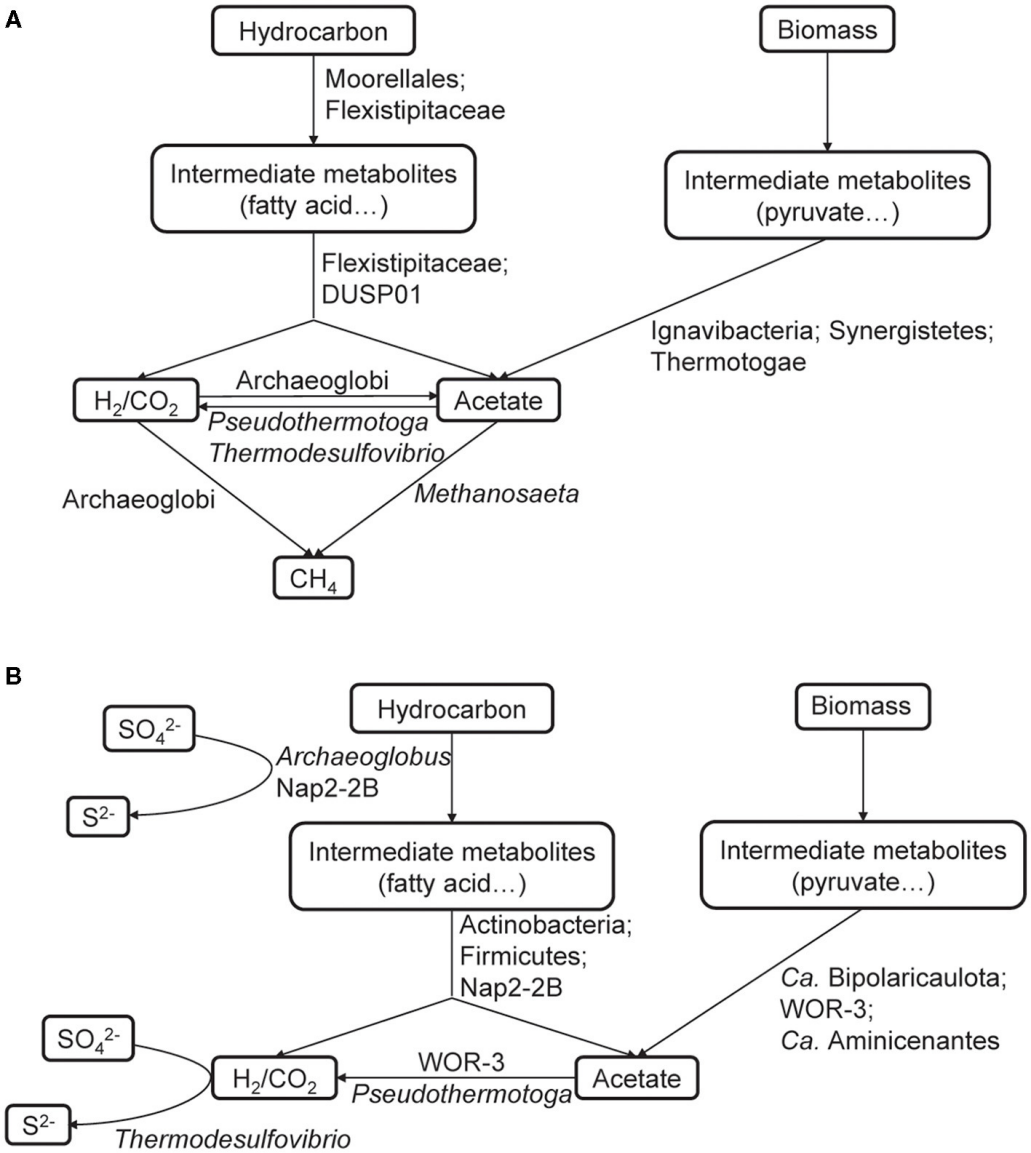
Reconstruction of potential metabolic interactions of the methanogenic **(A)** and sulfate-reducing **(B)** communities.

In anoxic habitats, hydrocarbons are usually fermented into intermediates such as acetate, CO_2_, and hydrogen. Due to thermodynamic limitations, many fermentation reactions do not yield sufficient energy to support growth at high concentrations of intermediates. Nevertheless, it becomes favorable when the intermediates are consumed by other organisms. This tightly coupled mutualistic relationship is called “syntrophy” (Sieber et al., 2012; Walker et al., 2012; Gieg et al., 2014). In consideration of the difference in electron acceptors, it is obvious that different hydrocarbon degraders and their partners were recovered from each enrichment culture. Moorellales and Flexistipitaceae participated in hydrocarbon degradation in the methanogenic community, whereas *Archaeoglobus* and Nap2-2B were the key players under sulfate-reducing conditions (Figure 4). Despite the variations in hydrocarbon degraders, we found that putative syntrophic interactions occurred in both enrichment cultures. Abundant fermentative bacteria and SAO bacteria were detected, including FUM_bin.31_Moorellales, FUM_bin.30_Ch128b, FUM_bin.26_Thermotogales, and FUM_bin.58_Thermodesulfovibrionales in the methanogenic communities, as well as SO4_bin.33_Desulfotomaculales, SO4_bin.1_UBA1063, SO4_bin.2_UBA1063, SO4_bin.18_Bipolaricaulales, and SO4_bin.25_Aminicenantales in the sulfate-reducing communities. These microbial groups could produce H_2_ and/or acetate through H_2_-evolving hydrogenases and/or AcdA, which were then supplied to methanogens (FUM_bin.88_Archaeoglobales and FUM_bin.65_Methanotrichales) or H_2_-utilizating SRP (SO4_bin.81_Thermodesulfovibrionales) (Figure 4). Thus, syntrophic biodegradation played a critical role in hydrocarbon degradation under both conditions.

In addition to thermodynamic syntrophic interdependencies, amino acid and vitamin auxotrophies have also been observed in methanogenic microbial assemblages. The exchange of amino acids and cofactors among members was crucial for the complex interdependencies among these anaerobic microorganisms, as previously described in hydrocarbon-degrading communities and acetate-degrading digestion ecosystems (Embree et al., 2015; Hubalek et al., 2017; Liu et al., 2018; Zhu et al., 2020). The two *Pseudothermotoga* MAGs (FUM_bin.26_Thermotogales and SO4_bin.10_Thermotogales), which we regarded as SAO bacteria, were incapable of energy-expensive tryptophan, phenylalanine and tyrosine synthesis but encoded genes for putative amino acid transporter systems (Supplementary Figure S3). We inferred that energy-inefficient SAO forced *Pseudothermotoga* to reduce the burden of amino acid biosynthesis. Previous studies have proposed that *Coprothermobacter proteolyticus* DTU632 relied on external amino acids for growth in an anaerobic digestion ecosystem due to the absence of efficient pathways for electron disposal (Zhu et al., 2020). These auxotrophies may be adaptations to nutrient and energy limitations (Hubalek et al., 2017). Interestingly, among the MAGs accounting for more than 1% of abundance in the sulfate-reducing community, the complete pathway of pantothenate biosynthesis was only found in Candidate division WOR-3 and *Ca*. Aminicenantes (Supplementary Figure S3). Pantothenate is a key precursor required for the biosynthesis of coenzyme A (CoA) and acyl carrier protein (ACP) (Zheng et al., 2004). Thus, pantothenate biosynthesis may give Candidate division WOR-3 and *Ca*. Aminicenantes an advantage for their continued retention in the community. However, the potential exchange of amino acids and cofactors among microbial communities from this study requires further confirmation.

### 3.5 Comparison of the two high-pressure-enriched communities to those under ambient pressure

To date, there have been several laboratory experiments demonstrating the methanogenic degradation of petroleum hydrocarbons under ambient atmospheric pressure at different temperatures (Gieg et al., 2010; Tan et al., 2015; Toth and Gieg, 2018; Chen et al., 2020; Ji et al., 2020; Liu et al., 2020a). These studies have revealed that Firmicutes, Synergistetes, and Thermotogae were often detected in communities incubated at a high temperature (55°C) as initial hydrocarbon degraders and/or fermentative bacteria (Gieg et al., 2010; Mbadinga et al., 2012; Chen J. et al., 2019; Xu et al., 2019). This finding was consistent with our present study, in which Firmicutes and Thermotogae were highly enriched (Supplementary Figure S1). In addition, these enrichment cultures, as well as oilfield investigation, indicated that SAO associated with hydrogenotrophic methanogenesis, mainly performed by Methanobacteriales, was the prevalent methanogenic pathway in high-temperature oil reservoirs (Dolfing et al., 2008; Li et al., 2017). However, in our study, the dominant hydrogenotrophic methanogen was FUM_bin.88_Archaeoglobales, which was less represented in previous methanogenic communities under ambient pressure. Recently, Verstraetearchaeota-type and divergent McrA were found in Archaeoglobi MAGs retrieved from the deep subseafloor and hot springs based on metagenome binning, indicating their capacity for methanogenic or alkanotrophic metabolism (Boyd et al., 2019; Wang et al., 2019b). Furthermore, Liu et al. revealed the *in situ* activity of hydrogen-dependent methylotrophic methanogenesis and heterotrophic fermentation within Archaeoglobi in a high-temperature oil reservoir through metatranscriptomic analysis (Liu et al., 2020b). However, as there are currently no representatives of the Archaeoglobi encoding the MCR complex, it is difficult to clarify the metabolic role of these Archaeoglobi in the subsurface biosphere.

In a recent study, Firmicutes, Thermotogae, Deltaproteobacteria, and Synergistetes were observed under sulfate-reducing and high-temperature conditions (Xiong et al., 2015). Accordingly, Firmicutes and Thermotogae were found to be abundant in our sulfate-reducing community, as well as in our methanogenic community. In addition, several SRPs, assigned to Deltaproteobacteria, Firmicutes, Nitrospira, Thermodesulfobacteria, Euryarchaeota, and Crenarchaeota, have been reported in oil reservoirs (Varjani and Gnansounou, 2017). Among them, thermophilic SRPs *Desulfotomaculum, Desulfonauticus, Thermodesulforhabdus, Thermodesulfobacterium, Thermodesulfovibrio*, and *Archaeoglobus* were more represented in high-temperature reservoirs (Singh and Choudhary, 2021; Zhou et al., 2023). In our sulfate-amended culture, *Archaeoglobus* was the most abundant, followed by other SRPs belonging to the candidate family Nap2-2B and *Thermodesulfovibrio*. Based on the abundance of Firmicutes, Thermotogae, and Archaeoglobi in the two communities, it is suggested that they possessed a strong adaptive ability to high-temperature and high-pressure conditions. In addition, Nobu et al. suggested that several uncultivated phyla (Hydrogenedentes, Marinimicrobia, Atribacteria, and Cloacimonetes) performed fermentative, syntrophic, and acetogenic catabolism in the TA-degrading methanogenic community (Nobu et al., 2015a). In other studies, *Ca*. Bipolaricaulota and *Ca*. Aminicenantes were found during the methanogenic conversion of petroleum hydrocarbons (Xu et al., 2019; Liu et al., 2020a). However, there were few reports about microorganisms from candidate phyla in the sulfate-reducing community. In our study, candidate phyla were found to be abundant and predicted to be involved in H_2_ and acetate metabolism. The detection of microbial dark matter members, such as hydrogenotrophic Archaeoglobaceae, *Ca*. Bipolaricaulota, Candidate division WOR-3, and *Ca*. Aminicenantes indicated that high-pressure incubation might be an effective way to simulate conditions closer to the *in situ* environment to expand our knowledge of unknown microorganisms.

## 4 Conclusion

In summary, we applied a metagenomics approach to analyze the microbial community structure and metabolic potential in methanogenic and sulfate-reducing enrichments under high-pressure conditions. In the methanogenic community, genes encoding FaeA-like proteins were detected in Moorellales and Flexistipitaceae. Hydrogenotrophic Archaeoglobaceae and acetoclastic *Methanosaeta* were dominant among the methanogens. On the other hand, *Archaeoglobus* and Nap2-2B were dominant in hydrocarbon degradation accompanying sulfate reduction. Candidate division WOR-3 and *Ca*. Aminicenantes accounted for a larger proportion in the sulfate-reducing community and produced pantothenate for other community members. Given the detection of hydrogenotrophic Archaeoglobaceae, *Ca*. Odinarchaeota, *Ca*. Methanosuratus, Candidate division WOR-3, and *Ca*. Aminicenantes in the two incubations, further studies are required to elucidate the interactions among these microbial dark matter members and the rest of the community. Additionally, the combination of incubation under *in situ* pressure conditions and metagenomic-/metatranscriptomic-based methods may provide more information on the microbial metabolic potential in petroleum reservoirs. These studies enriched our current knowledge of metabolic profiling of oil-degrading microbial communities under high-pressure conditions.

## Data availability statement

The datasets presented in this study can be found in online repositories. The names of the repository/repositories and accession number(s) can be found in the article/Supplementary material.

## Author contributions

JX: Software, Writing – original draft, Writing – review & editing. LW: Methodology, Writing – review & editing. WL: Resources, Writing – review & editing. XS: Resources, Writing – review & editing. YN: Supervision, Writing – review & editing. X-LW: Supervision, Writing – review & editing.
