# Supplementary material for: Metabolic profiling of petroleum-degrading microbial communities incubated under high-pressure conditions
Source: Front Microbiol. 2023 Dec 22;14:1305731. doi: 10.3389/fmicb.2023.1305731 (PMC10766756; doi:10.3389/fmicb.2023.1305731)
Supplement: Supplementary file 3 [file Data_Sheet_1.DOCX]

Supplementary Material

## Metabolic profiling of petroleum-degrading microbial communities incubated under high-pressure condition

Jinbo Xu^1, 2*^, Lu Wang^2*^, Weifeng Lv^2^, Xinmin Song^2^, Yong Nie^3#^, Xiao-Lei Wu^3, 4#^

^1^ School of Earth and Space Sciences, Peking University, Beijing 100871, PR China

^2^ State Key Laboratory of Enhanced Oil & Gas Recovery, Research Institute of Petroleum Exploration & Development, Beijing 100083, PR China

^3^ College of Engineering, Peking University, Beijing 100871, PR China

^4^ Institute of Ecology, Peking University, Beijing 100871, PR China

^#^Correspondence: [nieyong@pku.edu.cn](mailto:nieyong@pku.edu.cn), xiaolei_wu@pku.edu.cn

^*^ Jinbo Xu and Lu Wang contributed equally to this work.

**Supplemental Figure:**


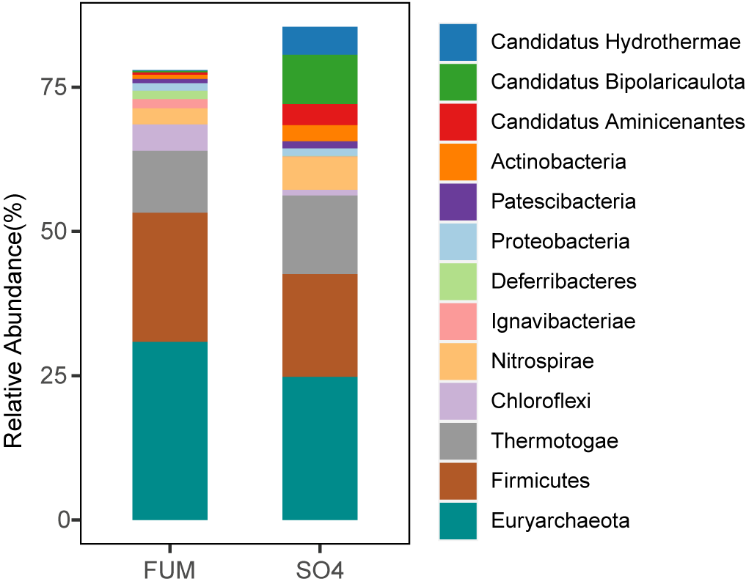


Fig. S1 The relative abundance of microbial taxa in the metagenomes.


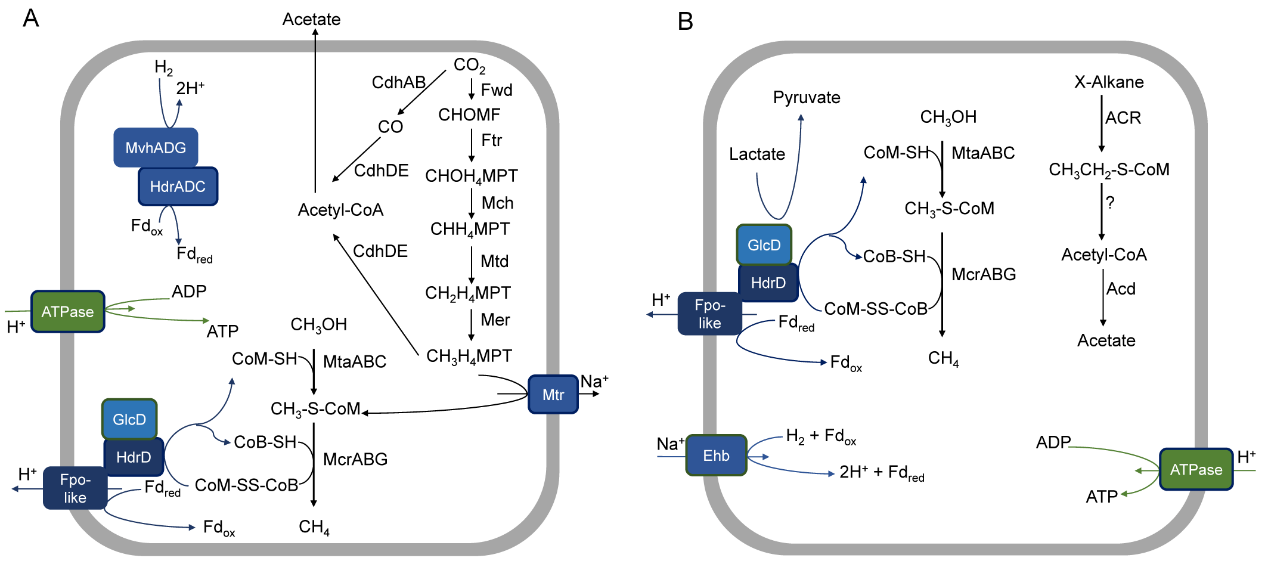


Fig. S2 Predicted methane or alkane metabolism in FUM_bin.88 (A) and FUM_bin.40 (B). A, Pathways for methylotrophic methanogenesis and hydrogenotrophic methanogenesis in FUM_bin.88 (o_Archaeoglobales, g_WYZ-LMO2). B, Pathways for H_2_-dependent methylotrophic methanogenesis and short-chain alkane oxidation in FUM_bin.40 (o_Methanomethylicales, g_*Methanosuratus*).


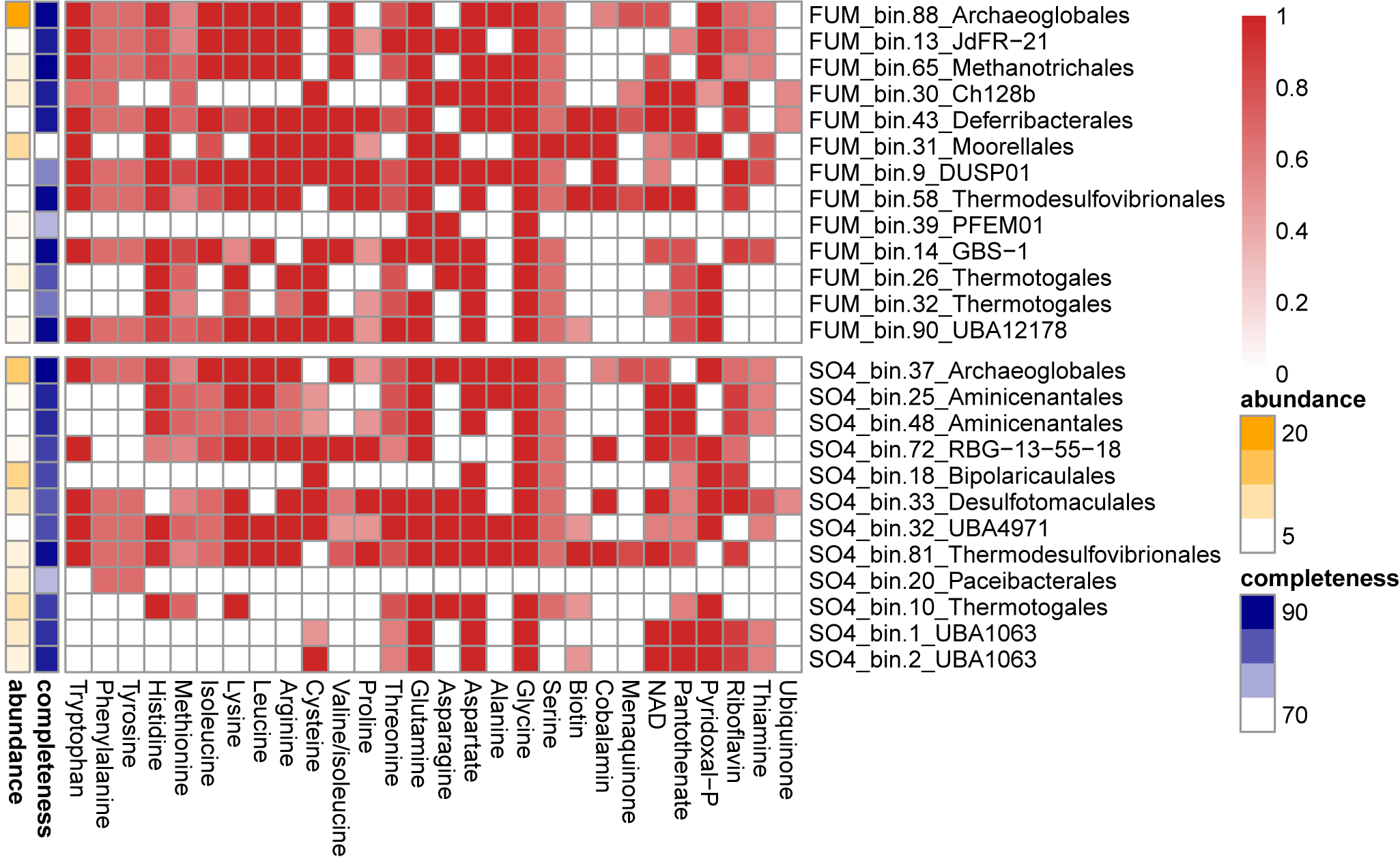


Fig. S3 Heatmap of amino acid and vitamin biosynthetic capabilities in the MAGs

**Supplemental Table:**

Table S1 Information of medium- to high-quality MAGs. The completeness, contamination, GC, N50 and size of MAG were evaluated by CheckM. The abundance was evaluated by CoverM. The taxonomy was annotated by GTDB-Tk.

Table S2 Metabolic pathways or genes analysis of medium- to high-quality MAGs.
